# PNPLA3(I148M) Inhibits Lipolysis by Perilipin-5-Dependent Competition with ATGL

**DOI:** 10.3390/cells12010073

**Published:** 2022-12-24

**Authors:** Hagen Roland Witzel, Inga Maria Gertrud Schwittai, Nils Hartmann, Sebastian Mueller, Jörn M. Schattenberg, Xue-Min Gong, Johannes Backs, Peter Schirmacher, Detlef Schuppan, Wilfried Roth, Beate Katharina Straub

**Affiliations:** 1 Institute of Pathology, University Medical Center Mainz, 55131 Mainz, Germany; 2 Department of Internal Medicine, Salem Medical Center, 69121 Heidelberg, Germany; 3 Metabolic Liver Research Program, I. Department of Medicine, University Medical Center, 55131 Mainz, Germany; 4 Institute of Experimental Cardiology, Heidelberg University Hospital, 69120 Heidelberg, Germany; 5 Institute of Pathology, University Medical Center Heidelberg, 69120 Heidelberg, Germany; 6 Translational Immunology, Research Center for Immune Therapy, University Medicine Mainz, 55131 Mainz, Germany; 7 Division of Gastroenterology, Beth Israel Deaconess Medical Center, Harvard Medical School, Boston, MA 02115, USA

**Keywords:** PNPLA3, perilipin, NAFLD, lipoloysis, lipid droplets, non-alcoholic steatohepatitis (NASH), alcoholic steatohepatitis (ASH), ballooned hepatocytes

## Abstract

The single nucleotide polymorphism I148M of the lipase *patatin-like phospholipase domain containing 3* (*PNPLA3*) is associated with an unfavorable prognosis in alcoholic and non-alcoholic steatohepatitis (ASH, NASH), with progression to liver cirrhosis and development of hepatocellular carcinoma. In this study, we investigated the mechanistic interaction of PNPLA3 with lipid droplet (LD)-associated proteins of the perilipin family, which serve as gatekeepers for LD degradation. In a collective of 106 NASH, ASH and control liver samples, immunohistochemical analyses revealed increased ballooning, inflammation and fibrosis, as well as an accumulation of PNPLA3–perilipin 5 complexes on larger LDs in patients homo- and heterozygous for PNPLA3(I148M). Co-immunoprecipitation demonstrated an interaction of PNPLA3 with perilipin 5 and the key enzyme of lipolysis, adipose triglyceride lipase (ATGL). Localization studies in cell cultures and human liver showed colocalization of perilipin 5, ATGL and PNPLA3. Moreover, the lipolytic activity of ATGL was negatively regulated by PNPLA3 and perilipin 5, whereas perilipin 1 displaced PNPLA3 from the ATGL complex. Furthermore, ballooned hepatocytes, the hallmark of steatohepatitis, were positive for PNPLA3 and perilipins 2 and 5, but showed decreased perilipin 1 expression with respect to neighboured hepatocytes. In summary, PNPLA3- and ATGL-driven lipolysis is significantly regulated by perilipin 1 and 5 in steatohepatitis.

## 1. Introduction

Fatty liver disease (FLD, novel synonym: steatotic liver disease/SLD), the accumulation of lipid droplets (LDs) in hepatocytes, is a steadily increasing health risk affecting more than one-third of the population in the western world. Chronic hepatitis C, alcohol abuse and metabolic syndrome are the main causes of hepatocyte steatosis (alcoholic/non-alcoholic fatty liver disease: AFLD/NAFLD) [1]. NAFLD is the most common liver disease in the western world, with a prevalence of 25% [2]. In some patients, bland steatosis progresses to steatohepatitis (NASH, in analogy to the term ASH) [3] and liver cirrhosis, which bears an increased risk of developing hepatocellular carcinoma (HCC). The histological criteria that distinguish steatohepatitis from bland steatosis include hepatocyte ballooning, commonly regarded as the hallmark of steatohepatitis, as well as lobular inflammation [4,5].

The most common and strongest genetic risk factor for steatotic liver diseases is a common missense variant of *PNPLA3* (patatin-like phospholipase domain-containing protein; rs738409), PNPLA3(I148M) [6,7,8]. To date, only few studies offer possible explanations for the underlying mechanism, suggesting that steatosis is triggered by the accumulation of PNPLA3(I148M) protein at LDs [9]. PNPLA3 is a lipase with weak hydrolytic activity towards glycerolipids, whereas the variant PNPLA3(I148M) shows an almost complete loss of enzymatic activity [10]. Interestingly, *Pnpla3* knockout mice do not develop steatosis [11,12]; therefore, it appears unlikely that loss of hydrolytic activity is a sufficient explanation for the induction of steatosis. Only the overexpression of PNPLA3(I148M) and not of PNPLA3 induced FLD [13]. PNPLA3(I148M) knockin strategies in mice showed no effects under normal diet, but when fed a high-sucrose diet, the mice developed FLD [14]. PNPLA3(I148M) showed no significant changes in transcript levels [14], but accumulated at the protein level, possibly via an altered proteasomal degradation of PNPLA3(I148M) [11,15]. Increased triacylglycerol synthesis [16] of the sequence variant or altered secretion of triacylglycerides (TAGs) from the liver have been suggested as causes [17,18]. The most compelling cause appears to be the replacement and sequestration of lipases (ATGL) and cofactors (ABHD5) involved in lipolysis [18]. Together with PNPLA3, ATGL (adipocyte triglyceride lipase, synonym: PNPLA2) belongs to the PNPLA family of proteins. ATGL is the rate-limiting lipase in lipolysis [19]. ATGL is regulated by its co-factor ABHD5 as well as perilipins [20], the most abundant LD-associated proteins.

LD-associated proteins of the perilipin-family play a crucial role in LD-biogenesis, -maintenance and -structure via interaction with the cytoskeleton [21], as well as during lipolysis mediated by an interaction of perilipin 1 with hormone-sensitive lipase (HSL) and of perilipin 5 with ATGL [20,22]. In humans, the perilipin-family comprises five members: perilipin 1 (PLIN1, perilipin), perilipin 2 (PLIN2, adipophilin), perilipin 3 (PLIN3, TIP47), perilipin 4 (PLIN4, S3-12) and perilipin 5 (PLIN5, MLDP, Oxpat). Perilipins have been shown to be an essential part of LDs, as *Plin1-* [23,24], *Plin2-* [25], and *Plin5-* [26] knockout mice lack subpopulations of LDs in certain cell types. In humans, heterozygous mutations of *PLIN1* lead to lipodystrophy, hypercholesterolemia and FLD [27], most probably via impaired perilipin 1-driven lipolysis in adipose tissue. In previous studies, it was demonstrated that perilipins are differentially expressed in hepatocyte steatogenesis [28] and neoplastic steatogenesis [29]. Perilipin 1 decorates LDs in chronic steatotic liver disease [30], whereas perilipin 3 and 5 localize to small LDs in microvesicular steatosis as observed in acute liver damage [31].

Since lipolysis requires the interaction of lipases with perilipins such as perilipin 1 and 5 to gain access to the LD-core, the present study was aimed to determine the interplay between perilipins and PNPLA3 in human liver in steatohepatitis in situ and to unravel the mechanism by which PNPLA3(I148M) aggravates SLD.

## 2. Materials and Methods

### 2.1. Tissues and Cell Culture

HEK293T, Huh7, and HepG2 cells, obtained from ATCC, were grown in DMEM (Gibco) supplemented with 10% fetal bovine serum (Sigma-Aldrich, St. Louis, MO, USA) and 1% of 10,000 U/mL penicillin, and 10 mg/mL streptomycin (Sigma) at 37 °C and 5% CO_2_. Cell lines were tested for mycoplasma contamination on regular basis.

Cryopreserved (snap-frozen) as well as formalin-fixed paraffin-embedded human tissue samples were obtained from the tissue biobank of the University Medical Center Mainz in accordance with the regulations of the tissue biobank and the ethics vote of Rhineland-Palatinate to BKS and HRW (title “Lipidtropfen-assoziierte Proteine und ihre Rolle in der Entstehung von alkoholischer und nicht-alkoholischer Steatohepatitis in Patienten mit häufig auftretenden PNPLA3-Polymorphismen”; 2018-13209). The collective of cryopreserved tissues included 15 liver specimens from patients with colorectal liver metastases taken from a location distant from the metastasis.

For immunohistochemistry, 59 formalin-fixed, paraffin-embedded liver biopsies of 23 patients with NASH, 19 patients with ASH, including 4 with alcoholic and metabolic (BASH) as well as 21 liver biopsies from control patients (normal liver, HCV; unclear cases, see Table 1) were used and compared to a collective of 47 ASH cases previously described [32]. These samples exhibited the *PNPLA3* genotypes 23× *C*/*C*, 17× *C*/*G*, and 7× *G*/*G*, respectively (study in accordance with the ethics committee of the University of Heidelberg (no: S280-2011).

### 2.2. Mouse Models

*ATGL* knockout mice were received from H. Haemmerle [19] and were kept in accordance with the regulations of the local ethics committee of Heidelberg (AZ 35-9185.81/G-72/16).

### 2.3. Protein Isolation

After washing cultured cells (10 cm plates) with PBS, cells were pelleted at 1000 rpm, resuspended in 600 µL RIPA buffer (50 mM TRIS-HCl, 15 mM EGTA, 100 mM NaCl, 1% (*v*/*v*) triton X-100, pH = 8.0) supplemented with 1:100 Halt™ protease and phosphatase inhibitor (Thermo Scientific^TM^, Waltham, MA, USA), and lysed with the tip of a sonicator for 10 s. The homogenate was then centrifuged at 12,000× *g* for 5 min. Protein concentration was determined by the Bradford protein assay (Bio-Rad).

Cryopreserved human tissue sections with a thickness of 5 μm were placed in tissue homogenizing kit CKMix tubes (Precellys) and then lysed with a Precellys 24 homogeniser 2 times at 6500 rpm for 20 s.

### 2.4. Antibodies

The antibodies and conditions are described in the Supplementary Materials, Materials and Methods.

### 2.5. Transfection

Transfection of HEK293T, Huh7, and HepG2 cells was performed as previously described [31,33].

### 2.6. Co-Immunoprecipitation

Co-immunoprecipitation was performed as previously described [33].

### 2.7. Immunofluorescence Microscopy

HEK293T, Huh7, and HepG2 cells were seeded on 13 mm (⌀) coverslips in 12-well plates, transfected the next day and fixed after an additional incubation period of 24 h or 48 h. After washing with PBS, cells were fixed for 10 min with 3.7% formaldehyde in PBS. Subsequently, cells were washed with PBS, permeabilized, and blocked with 10% FBS, 0.1% Triton X-100 in PBS at 37 °C. Cells were then incubated with the primary antibody in blocking solution at RT for 1 h. After a brief wash with 0.1% Triton X-100 in PBS, cells were either incubated with the secondary antibodies (Alexa Fluor^®^ 546 goat anti-mouse IgG (H + L), Alexa Fluor^®^ 635 goat anti-rabbit IgG (H + L), Alexa Fluor^®^ 635 goat anti-mouse IgG (H + L) or without (EGFP- and mRFP fusion proteins), the nuclear dye DAPI and the LD-dye BODIPY^TM^ 493/503 in 1% FBS, 0.1% Triton X-100 in PBS at RT for 1 h. After a final washing step with 0.1% Triton X-100 in PBS, cells were mounted with MOWIOL and images were taken with a Leica SP8 confocal microscope using either a 40× 1.30 NA Oil CS2 HC Plan Apo or a 63× 1.40 NA Oil CS2 HC Plan Apo objective operating at 25 °C. Both objectives were operated with Type F immersion liquid (Leica Microsystems). Leica Application Suite X was used to acquire the images. LD-size was then analysed with ImageJ (v.1.52n; https://imagej.net/downloads, accessed on 1 December 2022). For detailed instructions, please see the Supplementary Materials, Materials and Methods.

### 2.8. Image Analysis

Stained sections were digitised with a NanoZoomer 2.0 HT (Hamamatsu) at a magnification of 40× and the intensity of the staining was quantified using the digital image analysis software QuPath (v.0.1.2) [34]. To determine the area occupied by LDs in biopsies, the digitised images were analysed with the software Aperio ImageScope (v.12.2.2.5015, Leica Biosystems, Wetzlar, Germany). For detailed instructions, please see the Supplementary Materials, Materials and Methods.

### 2.9. Immunohistochemistry

Immunohistochemistry of formalin-fixed and paraffin-embedded liver biopsies was performed as previously described [28,29]. Stained sections were evaluated manually by an experienced hepatopathologist (BKS) and in parallel, digitally. Staining intensity was scored in accordance with the Allred Score (sum of intensity: 0 = negative, 1 = weak, 2 = moderate, 3 = strong staining, and percentage of positive cells: 0%: 0, <1%: 1, 1–10%: 2, 11–33%: 3, 34–66%: 4, 67–100%: 5, i.e., 0–8). In addition, steatosis, steatohepatitis, and fibrosis were graded [4,35]. Stained sections were digitalized with a NanoZoomer 2.0 HT (Hamamatsu) at a magnification of 40×, and the intensity of the staining was evaluated using the digital image analysis software QuPath (v.0.1.2) [34].

### 2.10. Transmission Electron Microscopy

Transmission electron microscopy of glutaraldehyde fixed liver biopsies was performed as previously described [28,29]. Images were taken on a JEOL JEM 1400 electron microscopy with a 4 k camera.

### 2.11. CRISPR/Cas

HEK293T cells did not harbour the PNPLA3(I148M) missense variant on either of the two alleles. Therefore, a previously described protocol [36] was used to generate PNPLA3(I148M) knockin cell lines with two designed guides and oligonucleotides ligated into the plasmid pSpCas9(BB)-2A-Puro (PX459) V2.0. Sequences of the guide oligonucleotides used for cloning were PNPLA3_G2_for 5′-CACCGCCTTCAGAGGCGTGGTAAGT-3′, PNPLA3_G2_rev 5′-AAACACTTACCACGCCTCTGAAGGC-3′, PNPLA3_G6_for 5′-CACCGGGGATAAGGCCACTGTAGAA-3′, PNPLA3_G6_rev 5′-AAACTTCTACAGTGGCCTTATCCCC-3′. Cells were transfected, genomic DNA was isolated and subjected to mutation analysis with HRMA and sequencing. Guide 2 (G2) and 6 (G6) showed the highest efficiencies and were therefore used to generate single cell clones. Selection of positively transfected cells was performed with 1 µg/mL puromycin for one week and cells were further seeded at a density of one cell per well in a 96-well plate. Single-cell clones were continuously expanded and screened for successful insertion of the single-stranded donor oligonucleotide (ssODN) in a high throughput procedure with the *HindIII* test digest when a 6-well plate size was reached and finally genotyped by sequencing.

PNPLA3_gtp_for 5′-AGGCCTTGGTATGTTCCTGC-3′, PNPLA3_gtp_rev 5′-TCCACCTTCCAGGGGTAACA-3′ were used as primers for genotyping. The sequence of the ssODN was PNPLA3_ssODN_HindIII 5′-CTATAACTTCTCTCTCCTTTGCTTTCACAGGCCTTGGTATGTTCCTGCTTCATGCCTTTTTATAGCGGACTTATCCCTCCAAGCTTCAGAGGCGTGGTAAGTCGGCTTTCTCTGCTAGCG-3′

### 2.12. Lipolysis/Steatogenesis

Lipolysis was induced by a combination of 10 µM forskolin with 500 µM IBMX for 1–3 h and compared with the vehicle (DMSO). Steatosis was induced with 240 µM oleic acid BSA complex for up to one week and compared with BSA treatment alone.

### 2.13. Quantitative PCR

Cryopreserved samples were collected in tissue homogenizing kit CKMix tubes (Precellys). A total of 1 mL TRI Reagent^TM^ Solution (Invitrogen) was added, and lysis was performed with a Precellys 24 homogeniser twice for 20 sec at 6500 rpm. RNA was isolated following the manufacturer’s instructions. RNA was reverse-transcribed into cDNA using the high-capacity cDNA reverse transcription kit (Applied Biosystems™), diluted, and used for real-time PCR (QuantStudio 3 Real-Time PCR System, Applied Biosystems). For sequences of real-time primers, please see the Supplementary Materials, Materials and Methods.

### 2.14. Statistical Analysis

Data were visualized as bar diagrams or scatter diagrams, each bar representing mean ± standard deviation. The statistical analysis was performed with GraphPad Prism v.9.4.0 (673; version 9.4.0 (673) for Windows, GraphPad Software, San Diego, California USA, www.graphpad.com. First, the data were tested for normal distribution using the Shapiro–Wilk normality test. If the data were normally distributed, a t-test was performed. If the standard deviations of the mean values of two groups differed significantly, Welch’s correction was also applied. If, on the other hand, the data were not normally distributed, the non-parametric Mann–Whitney test was used. *p*-values < 0.05 (*), *p* ≤ 0.001 (***), *p* ≤ 0.0001 (****) were considered significant, whereas *p*-values > 0.05 not significant (n.s.).

## 3. Results

### 3.1. Increased Inflammation and Fibrosis in Patients with PNPLA3(I148M)

Since the PNPLA3(I148M) polymorphism is associated with steatohepatitis and increased fibrogenesis in ASH and NASH patients, we analysed the histology as well as the expression pattern of the LD-associated proteins of the perilipin-family in liver biopsies of patients with steatotic liver disease with respect to PNPLA3 status. Immunohistochemistry against perilipins and in part together with PNPLA3 was performed with a total of 106 liver biopsies. In total, 23 NASH, 19 ASH (including 4 BASH-cases), 9 HCV and 8 control patients (Table 1) were analysed and compared with 47 ASH liver biopsies from a previous clinicopathological study [32].

**Figure 1:**
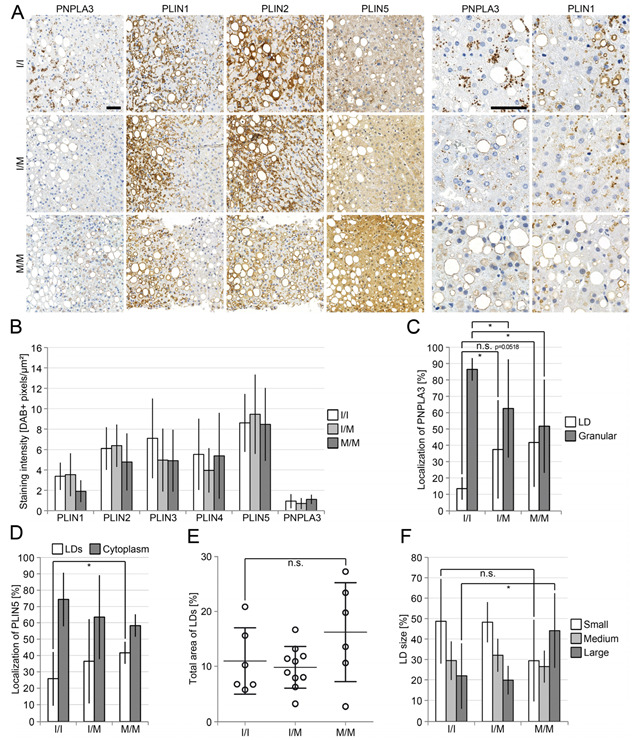
Reduced immunohistochemical perilipin 1, 2 and 3 staining in NASH-livers in homozygous PNPLA3(I148M)-carriers. (**A**) Immunohistochemical analysis of perilipin 1–5 and PNPLA3 in a collective of 23 liver biopsies from NASH-patients with known *PNPLA3*-status (I/I n = 7, I/M n = 10 and M/M n = 6). Hepatocytes strongly positive for PNPLA3 showed diminished perilipin 1-expression, especially in homozygous PNPLA3(I148M)-carriers. Scale bars: 50 μm; (**B**) analysis of the staining intensity (DAB-positive pixels/μm²) performed with the image analysis software QuPath. Livers of homozygous PNPLA3(I148M)-carriers showed a slight reduction in perilipin 1, 2 and 3-staining (*p* = 0.0561; *p* = 0.3529; *p* = 0.2904); (**C**) comparative scores of PNPLA3-localization to LDs (white) or granular structures (grey) in hepatocytes (0–4) demonstrated that LD-association of PNPLA3 was increased in heterozygous PNPLA3(I148M)-carriers (*p* = 0.0352) and PNPLA3-positive granules were reduced in homo- (*p* = 0.0299) as well as heterozygous carriers (*p* = 0.0352); (**D**) analysis of the localization of perilipin 5 (percentage of all stained cells). The presence of PNPLA3(I148M) favoured LD-association and simultaneously decreased cytoplasmic localization of perilipin 5; (**E**) determination of the area occupied by LDs in % as performed with ImageJ showed that the area occupied by LDs was increased in homozygous PNPLA3(I148M)-carriers (*p* = 0.2211); (**F**) analysis of LD-size and its percentage based on the total area of all LDs as performed with ImageJ. Compared with NASH-livers with PNPLA3 I/I status, homozygous PNPLA3(I148M)-carriers showed a reduced percentage of smaller (*p* = 0.132) and a significantly increased percentage of larger LDs (*p* = 0.0497), whereas the percentage of medium-size LDs did not change. All bars represent the mean ± SD. *p* < 0.05 (*); n.s. not significant.

**Figure A1:**
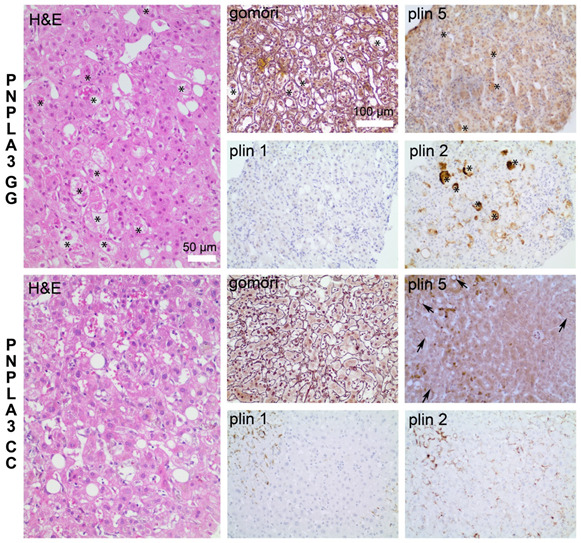
Immunohistochemistry of perilipins in liver biopsies of ASH patients with different PNPLA3-polymorphisms. Immunohistochemical images of perilipin 1, 2, 5, gömöri and H&E staining in a collective of 47 liver biopsies from ASH-patients with known *PNPLA3*-status. In *PNPLA3 G*/*G* genotype, ballooned cells (asterisks) are more frequently detected, with strong positivity for perilipin 2 and reduced staining for perilipin 1 when compared with the *PNPLA3 C*/*C* genotype. Arrows depict LD- and granular staining of perilipin 5. Scale bars: 50 µm (H&E) and 100 µm (gömöri and immunohistochemistry).

**Figure A2:**
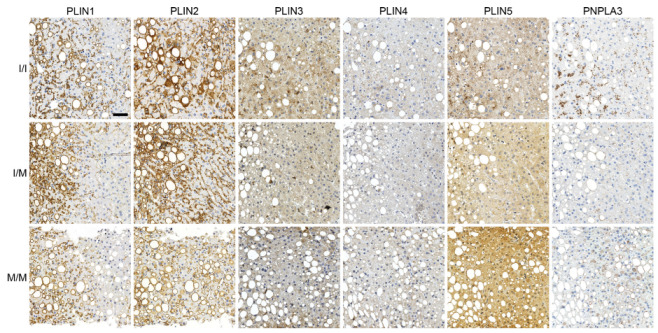
Reduced perilipin 1, 2 and 3 staining in livers of NASH patients carrying the homozygous PNPLA3(I148M)-polymorphism. Immunohistochemical analysis of perilipin 1-5 and PNPLA3 in a collective of 23 liver biopsies from NASH-patients with known PNPLA3-status as shown in Figure 1. Please note PLIN3 and PLIN4 immunohistochemical stainings, which are not shown in the main Figure 1A. Scale bar: 50 μm.

We could thereby recapitulate a positive correlation of PNPLA3(I148M) with steatosis, inflammation, the amount of microgranulomas, ballooning and fibrogenesis [32]. Generally, steatosis was strongly highlighted by perilipin 1 and 2 staining at LDs, whereas perilipins 3, 4 and 5 showed predominant cytoplasmic and only faint LD-staining. There was no significant difference in the general intensity of perilipin stainings between liver biopsies of different *PNPLA3* genotypes (Figure 1A,B, Figure A1 and Figure A2, Table 2.

**Figure 2:**
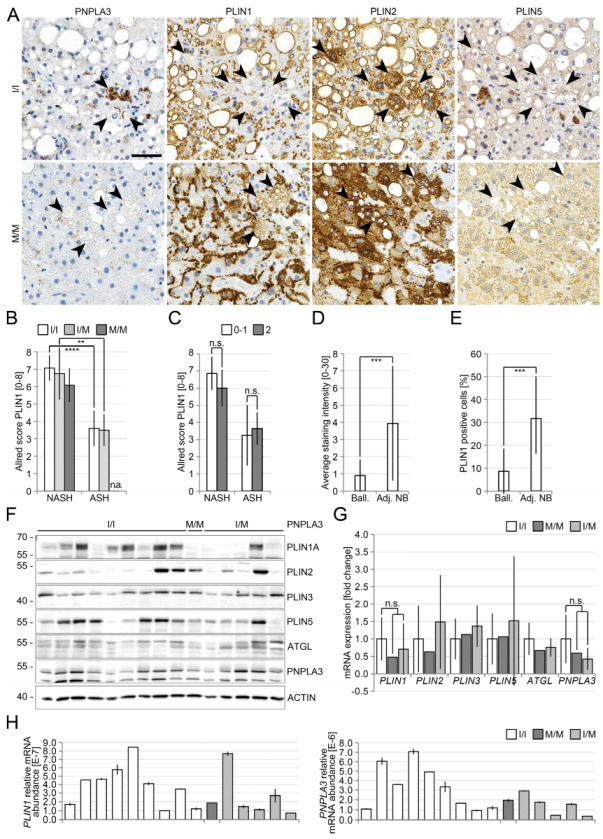
Expression pattern of PNPLA3 and perilipins in ballooned hepatocytes in ASH and NASH livers in situ. (**A**) Immunohistochemical analysis of PNPLA3, perilipin 1, 2 and 5 in ballooned hepatocytes (arrowheads) of either PNPLA3 I/I-or PNPLA3 M/M-carriers with NASH. Perilipin 1 staining was reduced in ballooned hepatocytes when compared to neighbouring hepatocytes; (**B**) perilipin 1 (PLIN1) was stained and scored in 23 NASH and 9 ASH liver specimens according to staining intensity and percentage of cells using Allred score and presented as averages with standard deviation; (**C**) perilipin 1 staining is reduced in livers of patients with ASH when compared to NASH, independent of the presence of ballooning. (**D**) Average staining intensity of perilipin 1 in ballooned hepatocytes (n = 15). (**E**) Average percentage of perilipin 1-positive ballooned hepatocytes (n = 15), when compared to neighbouring hepatocytes. Perilipin 1 is markedly reduced in ballooned hepatocytes. (**F**) Immunoblot analysis of perilipin 1, 2, 3 and 5, ATGL and PNPLA3 in a collective of 15 livers with different PNPLA3-status (I/I n = 4, M/M n = 9, I/M n = 2). Actin served as loading control. Perilipin 5 was slightly reduced in hetero- and homozygous carriers of the PNPLA3-polymorphism. (**G**) Real-time PCR analysis of *PLIN1*, *2*, *3* and *5*, *ATGL* and *PNPLA3* for the same samples shown in A. *PLIN1*- and *PNPLA3*-expression was reduced in homo- and heterozygous carriers of the PNPLA3-polymorphism, without reaching significance (*p* = 0.3416; *p* = 0.0931). Expression was normalized to I/I. Bars represent the mean of all samples with the same *PNPLA3* genotype ± SD. T-test for unpaired samples was performed in B. n.s. not significant. (**H**) Real-time PCR analysis of *PLIN1* and *PNPLA3* is shown in B and separated according to the individual samples and *PNPLA3* genotypes. Scale bar: 50 μm. All bars represent the mean ± SD. *p* < 0.01 (**); *p* ≤ 0.001 (***); *p* ≤ 0.0001 (****); n.s. not significant; Ball. = ballooned; Adj. NB = adjacent not ballooned.

However, strikingly, heterozygous PNPLA3(I148M)-carriers showed a weak, and homozygous carriers an even stronger localization of PNPLA3 at LDs in zones 1 and 2 of the liver which were negative or only weakly positive for perilipin 1 (Figure 1A,C), whereas in patients with PNPLA3(I148), PNPLA3 appeared almost exclusively in granular structures. In liver biopsies of PNPLA3(I148M)-carriers, perilipin 5 was more frequently localized at LDs and less in the cytoplasm (Figure 1D). The degree of steatosis was slightly higher in homozygous PNPLA3(I148M)-carriers (Figure 1E), accompanied by a significant increase in LD-size (Figure 1F). Ballooned hepatocytes were more frequently found in PNPLA3(I148M) carriers (Figure A1), and were strongly positive for perilipin 2 in both NASH and ASH livers irrespective of the *PNPLA3* genotype. These hepatocytes were also positive for PNPLA3 and perilipin 5; however, negative or only mildly positive for perilipin 1 (Figure 2A–C). In contrast, adjacent non-ballooned hepatocytes showed a significantly higher perilipin 1 expression (Figure 2A,D,E).

### 3.2. Reduced Perilipin 1 Expression in PNPLA3(I148M)-Hepatocytes In Situ

In order to investigate the underlying molecular mechanisms of PNPLA3 action, we examined the expression of PNPLA3 and perilipins at the protein and transcript level. Western blot analysis of 15 non-neoplastic livers of patients resected for colorectal metastases showed no obvious association between *PNPLA3*-genotype and the amount of PNPLA3-protein (Figure 2F). Perilipin 5 was slightly and perilipin 1 strongly reduced in livers of hetero- and homozygous carriers of the PNPLA3-polymorphism (Figure 2F). Real-time PCR analysis of the same samples showed reduced levels of *PLIN1* and *PNPLA3* mRNA in hetero- and homozygous carriers of the PNPLA3-polymorphism (Figure 2G,H). Interestingly, in the collective of 47 ASH patients, *PLIN5* mRNA was significantly downregulated, whereas PLIN1 was only mildly downregulated in liver biopsies of patients with PNPLA3(I148M) without reaching significance, PLIN2 and PLIN 3 mRNA levels were not significantly altered [32]. Similar changes were observed on a protein level using immunohistochemistry.

Our findings confirm our histological data and suggest a regulatory link between PNPLA3 and perilipin 1 and 5 on the protein and mRNA level, respectively, in human livers in situ.

### 3.3. Opposing Effects of Perilipin 1 and 5 on the Recruitment of PNPLA3 to LDs

**Figure 3:**
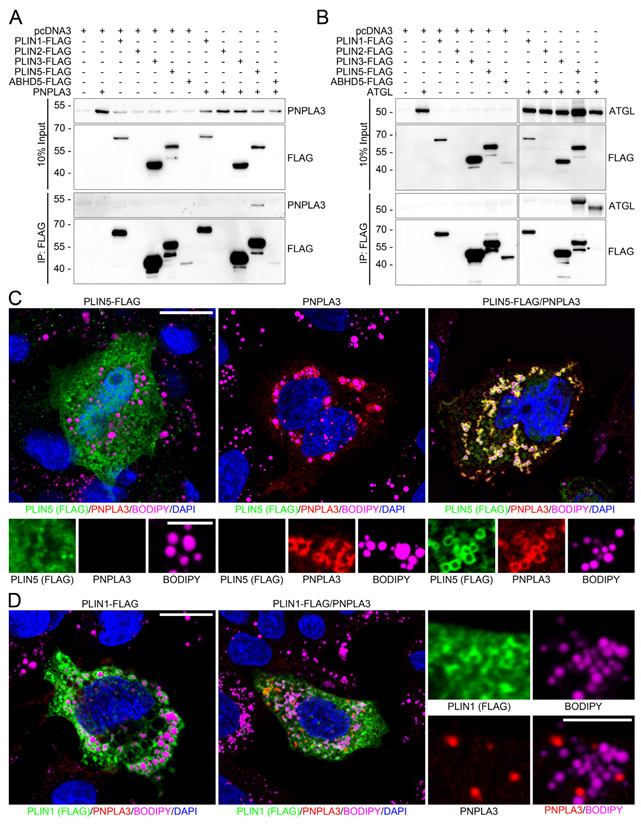
Opposing effects of perilipin 1 and 5 on the recruitment of PNPLA3 to LDs. (**A**,**B**) Immunoblot analysis of PNPLA3 in co-immunoprecipitates of FLAG-tag in HEK293T cells transfected with PNPLA3 or ATGL alone or together with FLAG-tagged PLIN1, 2, 3, 5 or ABHD5. PNPLA3 co-immunoprecipitates only with perilipin 5 (PLIN5), but not with other perilipins, whereas ATGL co-immunoprecipitates with perilipin 5 and ABHD5. The asterisk indicates a weak ABHD5-signal shifted in height. Molecular weight in kDa is given on the left. (**C**,**D**) Immunofluorescence microscopy of HepG2 cells transfected with FLAG-tagged perilipin 5 (green) or perilipin 1 (green) alone or with PNPLA3 (red) (LDs: BODIPY, violet; nuclei: DAPI, blue). Cells co-expressing PNPLA3 and perilipin 5 show strong colocalization at LDs. Co-expression of PNPLA3 and perilipin 1 shows granular cytoplasmic localization of PNPLA3 apart from LDs. Scale bars: 15 μm, and 5 μm for magnified images.

**Figure A3:**
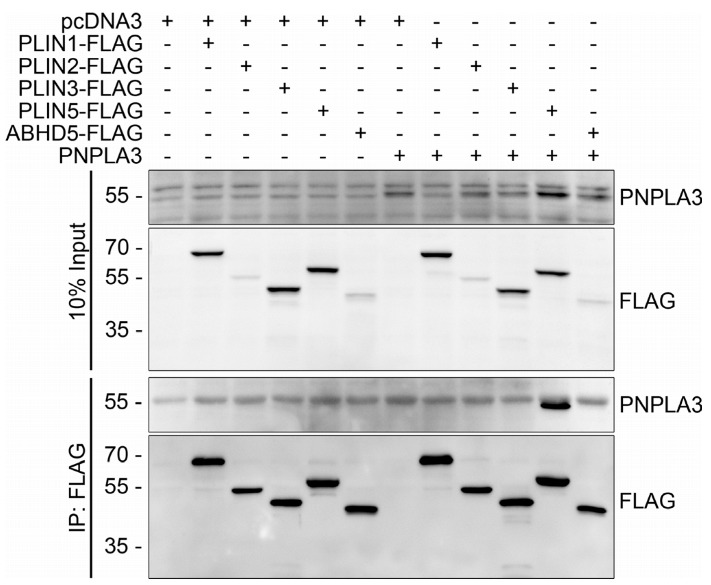
Co-precipitation of PLIN5 with PNPLA3 in the human hepatoma *cell* line Huh7. Immunoblot analysis of PNPLA3 in co-immunoprecipitates of FLAG-tag in Huh7 cells transfected with PNPLA3 alone or together with FLAG-tagged PLIN1, 2, 3, 5 or ABHD5. As already shown for HEK293T cells, PNPLA3 co-immunoprecipitates only with perilipin 5 (PLIN5), but no other perilipin. Molecular weight in kDa is given on the left.

**Figure A4:**
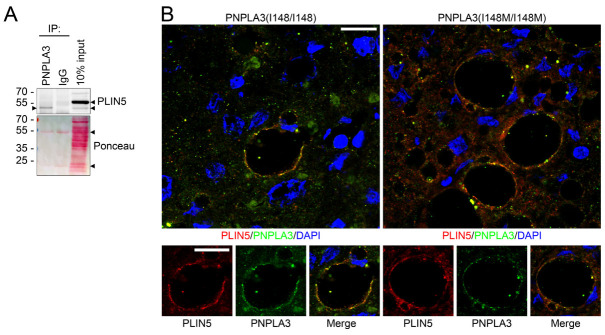
Validation of PLIN5-PNPLA3 complex formation in situ. (**A**) Immunoblot analysis of PLIN5 in co-immunoprecipitates performed with PNPLA3 antibody in post-nuclear lysates of human liver with PNPLA3(I148/I148). Only the lower migrating form of PLIN5 at approximately 52 kDa, but not the dominant full-length protein at approximately 55 kDa co-immunoprecipitates with PNPLA3. Ponceau staining of the same membrane shows that the same amount of antibody (PNPLA3, IgG) was used for IP. Arrows indicate the heavy and light chain of the antibodies. Molecular weight in kDa is given on the left. (**B**) Immunofluorescence microscopy of perilipin 5 (PLIN5, red) and PNPLA3 (green) of liver cryosections with different PNPLA3 status (nuclei: DAPI, blue). PLIN5 and PNPLA3 show colocalization at LDs. Scale bars: 15 μm.

Since perilipin 5 physically interacts with ATGL, the protein sharing the highest similarity with PNPLA3 [37], we hypothesized that perilipin 5 may form complexes with ATGL and PNPLA3. Therefore, PNPLA3 was overexpressed either in HEK293T or Huh7 cells alone and together with perilipin 1, 2, 3, 5, as well as ABHD5. In co-immunoprecipitation analyses, only perilipin 5 coprecipitated together with PNPLA3 in cell culture (Figure 3A and Figure A3) and in human liver (Figure A4) indicative of a direct or indirect interaction.

**Figure A5:**
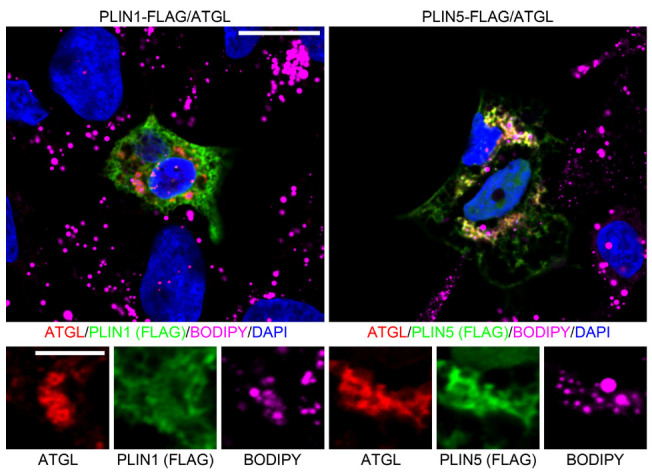
Perilipin 1 does not displace ATGL from lipid droplets (LDs). Immunofluorescence microscopy of perilipin 1 (PLIN1, green, left) or perilipin 5 (PLIN5, green, right) with ATGL (red) in transfected HepG2 cells (LD: BODIPY, violet, nuclei: DAPI, blue). HepG2 cells were transfected with ATGL together with FLAG-tagged PLIN1 or PLIN5. Perilipin 1 did not displace ATGL at LDs as discovered for the closely related PNPLA3. Co-expression of ATGL and PLIN5 led to strong colocalization at LDs in a comparable manner as for PNPLA3 and PLIN5. Scale bars: 15 μm, and 5 μm for magnified images.

Repetition of the in vitro studies with ATGL revealed interactions of ATGL with perilipin 5 and ABHD5 (Figure 3B) as already shown for skeletal muscle [22]. In immunofluorescence microscopy of transfected HepG2 cells (Figure 3C,D), perilipin 5 exhibited cytoplasmic as well as weak LD-localization and colocalized with PNPLA3 which was almost entirely LD-associated (Figure 3C). Co-expression of PNPLA3 and perilipin 5 resulted in a translocation of perilipin 5 from cytoplasmic to LD-bound localization and a strong colocalization of both proteins at LDs (Figure 3C) as observed for perilipin 5 with ATGL (Figure A5). This was confirmed by the colocalization of PNPLA3 with perilipin 5 at the same LDs in human liver cryosections in situ (Figure A4B) but not with other perilipins.

As shown for constitutive perilipins, perilipin 1 localized to LDs when overexpressed in cells (Figure 3D). Surprisingly, co-expression of PNPLA3 and perilipin 1 led to a complete displacement of PNPLA3 from LDs to granular cytoplasmic structures (Figure 3D). This mechanism appears to be specific for PNPLA3 since perilipin-1-induced displacement from LDs was not observed for ATGL (Figure A5). Perilipin 1 may thus provide a mechanism to regulate PNPLA3-binding to LDs.

### 3.4. PNPLA3 Interacts with ATGL, the Rate-Limiting Enzyme in Lipolysis, in a Perilipin 5-Dependent Manner

**Figure 4:**
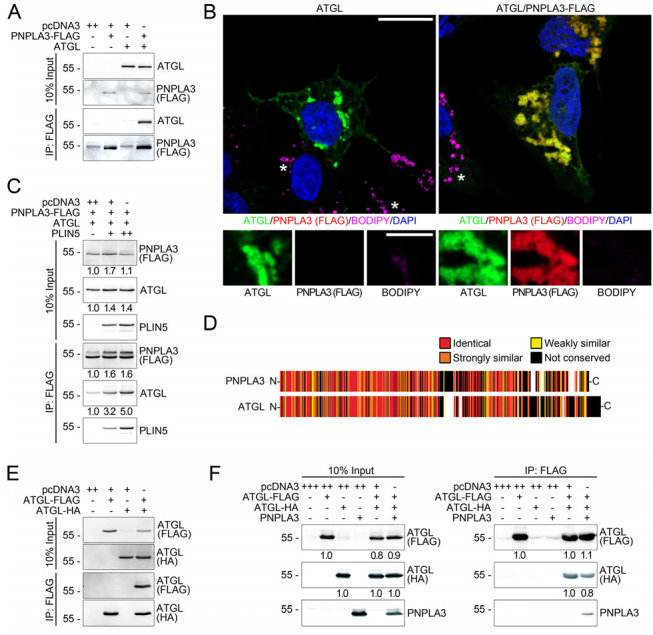
PNPLA3 interacts with ATGL in a perilipin 5-dependent manner. (**A**) Immunoblot analyses of ATGL and PNPLA3 in co-immunoprecipitates of FLAG-tagged PNPLA3 in HEK293T cells transfected with FLAG-tagged PNPLA3 alone and/or together with ATGL shows that PNPLA3 interacts with ATGL. (**B**) Immunofluorescence microscopy of HepG2-cells transfected with FLAG-tagged ATGL (green) alone or together with PNPLA3 (red). LDs: BODIPY, violet; nuclei: DAPI, blue. PNPLA3 and ATGL strongly colocalize at smaller sized LDs. Asterisks indicate non-transfected cells that exhibit LDs of detectable size. Scale bars: 15 μm, magnified images: 5 µm. (**C**) Immunoblot analysis of PNPLA3, ATGL and perilipin 5 (PLIN5) in co-immunoprecipitates of FLAG-tagged PNPLA3 in HEK293T cells transfected with FLAG-tagged PNPLA3, constant amounts of ATGL together with increasing amounts of perilipin 5 (PLIN5). The interaction of PNPLA3 with ATGL is dramatically increased by perilipin 5 in a dose-dependent manner. (**D**) Schematic representation of an alignment of PNPLA3 with ATGL shows a high degree of similarity. (**E**) Immunoblot analysis of ATGL (HA and FLAG) in co-immunoprecipitates of FLAG-tagged ATGL in HEK293T cells transfected with FLAG-tagged ATGL alone and together with HA-tagged ATGL shows that FLAG-tagged ATGL interacts with HA-tagged ATGL. (**F**) Immunoblot analysis of ATGL (HA and FLAG) and PNPLA3 in co-immunoprecipitates of FLAG-tagged ATGL in HEK293T cells transfected with FLAG-tagged ATGL alone and together with HA-tagged ATGL in the presence or absence of PNPLA3 shows that PNPLA3 favours PNPLA3-ATGL heterodimerization and disrupts ATGL-homodimerization. Molecular weight (kDa) is given on the left.

Since PNPLA3 and ATGL both localized to LDs (Figure 3C and Figure A5), we carried out studies to determine whether there was a possible interaction. In addition to a co-precipitation of ATGL with PNPLA3 (Figure 4A), both proteins also strongly colocalize to LDs (Figure 4B), suggesting the role of PNPLA3 in regulating ATGL.

As perilipin 5 was the only perilipin shown to co-precipitate with PNPLA3 and ATGL (Figure 4A), the impact of perilipin 5 on the interaction between PNPLA3 and ATGL was examined. In the absence of perilipin 5, PNPLA3 and ATGL interacted only weakly, whereas the presence of perilipin 5 led to a strong increase in PNPLA3 and ATGL interaction in a dose-dependent manner (Figure 4C). Phylogenetically, ATGL and PNPLA3 are the most closely related proteins within the PNPLA-family having 37% identical and a high degree of similar amino acids (Figure 4D). Therefore, we hypothesized that ATGL may also form homodimers. This was confirmed by ATGL-ATGL-complexes detected by co-immunoprecipitation using differently tagged ATGL-constructs (Figure 4E). However, small amounts of PNPLA3 were able to displace ATGL from the ATGL-ATGL-complexes (Figure 4F). Therefore, PNPLA3 may compete with ATGL providing a key mechanism to how PNPLA3 may interfere with the lipid metabolism.

### 3.5. PNPLA3(I148M) Negatively Regulates ATGL Activity

ATGL catalyzes the first step in the hydrolysis of TAGs [38,39,40]. Overexpression of ATGL leads to significantly smaller LDs, whereas downregulation by RNA-interference results in larger LDs [38].

**Figure 5:**
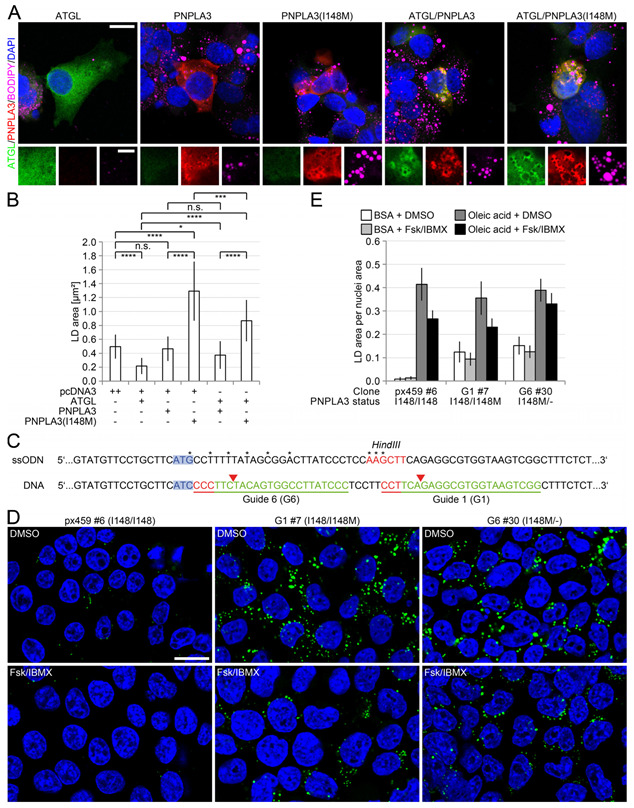
PNPLA3(I148M) negatively regulates ATGL-activity. (**A**) Immunofluorescence analysis of ATGL and PNPLA3 in HepG2 cells transfected with empty vector (n = 25), ATGL (n = 23), PNPLA3 (n = 12), and PNPLA3(I148M) (n = 16) alone or with ATGL in combination with either PNPLA3 (n = 14) or PNPLA3(I148M) (n = 17). Transfection of ATGL (green) led to a complete loss of LDs (LDs: BODIPY, violet; nuclei: DAPI, blue), whereas transfection of PNPLA3 (red) did not significantly affect LD size. Expression of PNPLA3(I148M) (red) leads to significantly larger LDs compared to cells transfected with PNPLA3. The expression of PNPLA3 together with ATGL leads to significantly larger LDs compared to cells transfected with ATGL. This effect is even higher in cells co-transfected with PNPLA3(I148M) and ATGL. (**B**) Quantification of LD-size in HepG2 cells shown in (**A**). The values are the results of the quantification of 3 independent experiments. (**C**) Schematic representation of the CRISPR/Cas strategy to introduce the PNPLA3(I148M) in HEK293T cells. Two guide RNAs (G1, G6) were designed and validated. *Protospacer* adjacent motif (PAM) sequence indicated in red, protospacer in green. Red arrows indicate Cas9 cleavage sites. Relevant target triplets are highlighted in light blue. Asterisks show silent mutations inserted to prevent re-cutting after successful insertion of the single-stranded donor oligonucleotide (ssODN). A *HindIII* site was inserted for screening reasons. (**D**) Induction of lipolysis in all three cell lines (I148/I148, I148/I148M, I148M/-) seeded at low density to prevent excessive LD-formation. (**E**) Repetition of the experiment in the presence and absence of a one-week steatogenic oleic acid treatment and quantification of LDs. As an example, the quotient between the total area of LDs and the total area of cell nuclei is demonstrated. Scale bars: 15 μm and 5 μm for magnified images. All bars represent the mean ± SD. *p* < 0.05 (*); *p* ≤ 0.001 (***); *p* ≤ 0.0001 (****); n.s. not significant.

**Figure A6:**
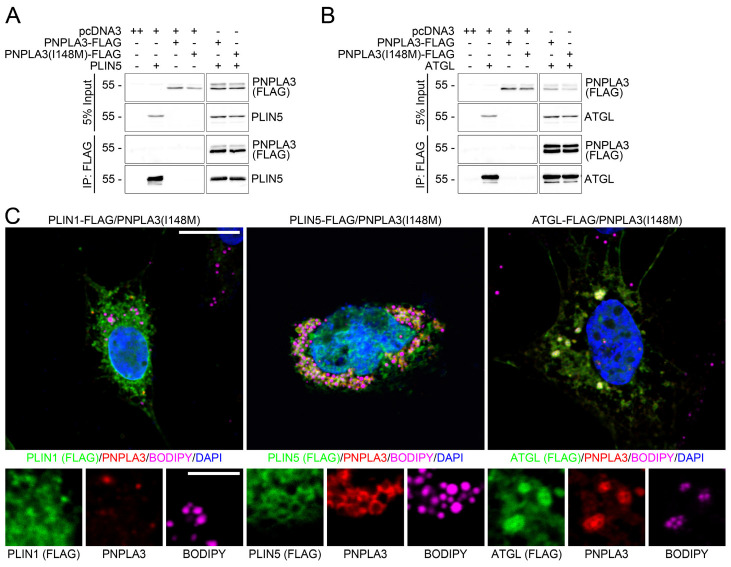
PNLA3 and PNPLA3(I148M) show identical interaction properties at the protein level and localization effects. Immunoblot analysis of PNPLA3, perilipin 5 (FLAG) and ATGL of Co-IP experiments of FLAG-tagged PNPLA3 and PNPLA3(148M). HEK293T cells were transfected with PNPLA3 or PNPLA3(I148M) alone and together with either FLAG-tagged PLIN5 (**A**) or ATGL (**B**). PNPLA3(I148M) showed an equal interaction behavior with PLIN5 or ATGL compared to PNPLA3. Molecular weight (kDa) is given on the left. (**C**) Immunofluorescence microscopy of perilipin 1 (PLIN1, green, left), perilipin 5 (PLIN5, green middle), or ATGL (green, right) with PNPLA3(I148M) (red) in transfected HepG2 cells (LDs: BODIPY, violet; nuclei: DAPI, blue). HepG2 cells were transfected with PNPLA3(I148M) alone or together with FLAG-tagged PLIN1, PLIN5, or ATGL and subsequently stained with antibodies against PNPLA3 and the FLAG epitope. Both the effects of PNPLA3(I148M) on the localization of perilipins and the effects of perilipins on PNPLA3(I148M) were indistinguishable from PNPLA3. Scale bars: 15 μm and 5 μm for magnified images.

**Figure A7:**
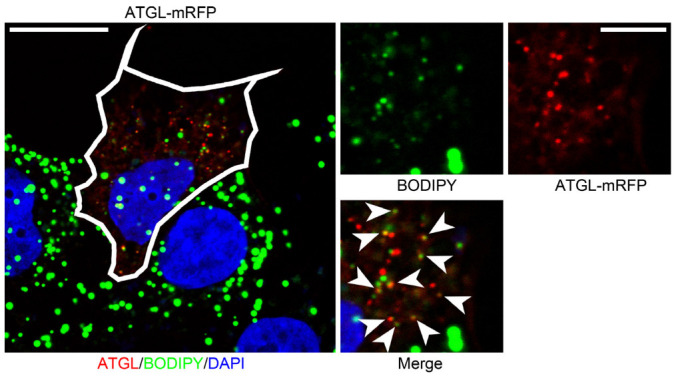
ATGL-positive foci colocalize with LDs. Immunofluorescence analysis of ATGL-positive foci (red) in transfected HepG2 cells. HepG2 cells were transfected with mRFP-tagged ATGL (red; nuclei: DAPI, blue). The majority of ATGL-positive foci colocalize with small residual LDs (BODIPY: green). Arrowheads indicate residual ATGL/BODIPY double-positive foci. Scale bars: 15 μm and 5 μm for magnified images.

When compared to wild-type PNPLA3, PNPLA3(I148M) did not show an altered interaction with perilipin 5 and ATGL or an altered localization in vitro (Figure A6). PNPLA3(I148M) leads to the reduced hydrolysis of fatty acids and accumulation of TAGs [11], yet this does not sufficiently explain the increased fat deposition and steatohepatitis in livers of I148M-carriers [17]. Therefore, we investigated whether ATGL-activity may be modulated by the physical interaction with PNPLA3. Overexpression of ATGL resulted in a significant reduction in the average LD size (see also Figure A7). In contrast to overexpression of PNPLA3, overexpression of PNPLA3(I148M) led to a significant increase in the average LD-size (Figure 5A,B). In contrast, lack of ATGL as in ATGL-deficient mice of 8 weeks of age demonstrated lack of ATGL, but not yet significant changes in expression of perilipins 2–5, PNPLA3 and ABHD5 as measured by immunoblot. ATGL-deficient mice at 12–13 weeks of age demonstrated microvesicular steatosis of the liver shortly before cardiac death, with increased expression of perilipin 2 and slight downregulation of perilipin 5, as measured by immunohistochemistry. Prominent microvesicular steatosis in ATGl-deficient mice of that age was also confirmed by transmission electron microscopy, which was not detected in wild-type mice of the same age. Due to early cardiac death of ATGL-deficient mice at about 12–13 weeks, older ages could not be analysed (Figure A9).

Co-expression of ATGL and PNPLA3 led to a significant increase in LD-size compared to cells expressing ATGL alone, which was even higher in the presence of PNPLA3(I148M) suggesting a negative effect on the lipolytic activity of ATGL.

**Figure A8:**
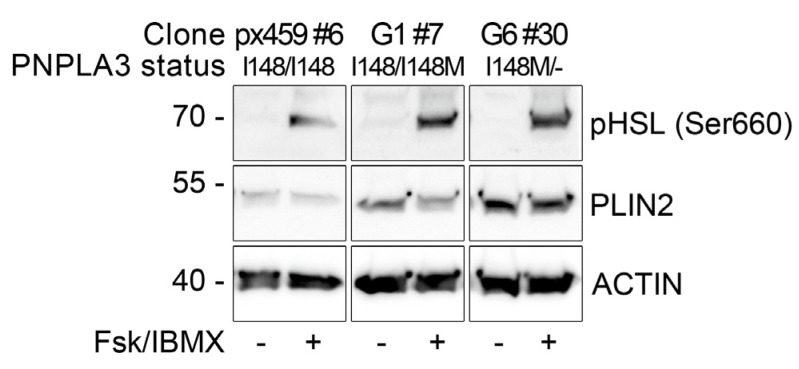
Induction of lipolysis in CRISPR/Cas single cell clones. Immunoblot analysis of phospho-HSL and perilipin 2 in CRISPR/Cas single cell clones after 1 h induction of lipolysis with 10 µM forskolin and 500 µM IBMX. Induction of lipolysis was indicated by a substantial increase in phosphorylated HSL (Ser600, pHSL) in all clones analyzed. Note that unmodified cells of origin (px459 #6) negative on both alleles for PNPLA3(I148M), showed the lowest expression for perilipin 2 (PLIN2), whereas higher levels were observed in the PNPLA3(I148M) heterozygous clone (G1 #7), and the highest PLIN2 levels in the clone carrying the PNPLA3(I148M) missense variant on one allele and on the other a knockout for PNPLA3. Molecular weight (kDa) is given on the left.

**Figure A9:**
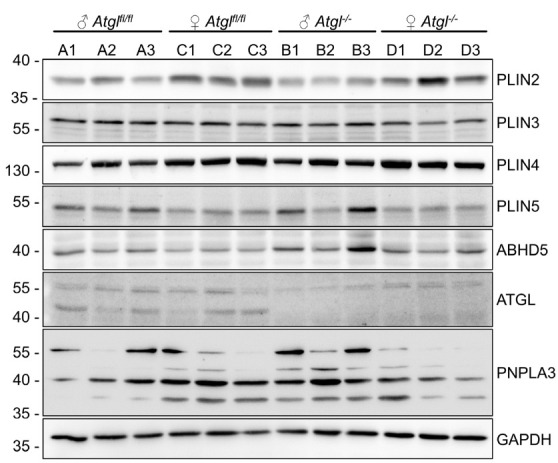
Perilipin 2-5, ATGL, and PNPLA3 in *ATGL* knockout mice of 8 weeks age. Immunoblot analyses of perilipin 2, 3, 4, and 5 (PLIN2, 3, 4, 5), ABHD5, ATGL, and PNPLA3 from whole cells liver lysates of floxed or *ATGL* knockout mice. Three mice per group were analyzed. GAPDH served as loading control. Molecular weight in kDa is shown on the left side.

To further investigate the negative effect of PNPLA3(I148M) on lipolysis, PNPLA3(I148M) knockin cells were generated (Figure 5C). Unmodified PNPLA3-control cells formed very few small LDs when seeded at a low density (Figure 5D,E). In PNPLA3(I148M)-heterozygous cells however, or cells carrying PNPLA3(I148M) and a knockout of one *PNPLA3* allele, LDs were significantly larger (Figure 5E). Following steatogenic treatment with oleic acid, all three cell lines showed comparable increases in LD size (Figure 5E and Figure A8). Lipolysis induced by forskolin and IBMX reduced the LD-size differently among cell lines, which was highest in control cells, and less pronounced in heterozygous I148/I148M cells. No significant difference was observed in I148M/- cells treated with vehicle or lipolytic stimuli, suggesting that the missense variant had the highest negative impact on cAMP-mediated lipolytic activity (Figure 5D,E).

### 3.6. Perilipin 1 Displaces PNPLA3 from the ATGL Complex and Drives ATGL-Mediated Lipolysis

**Figure 6:**
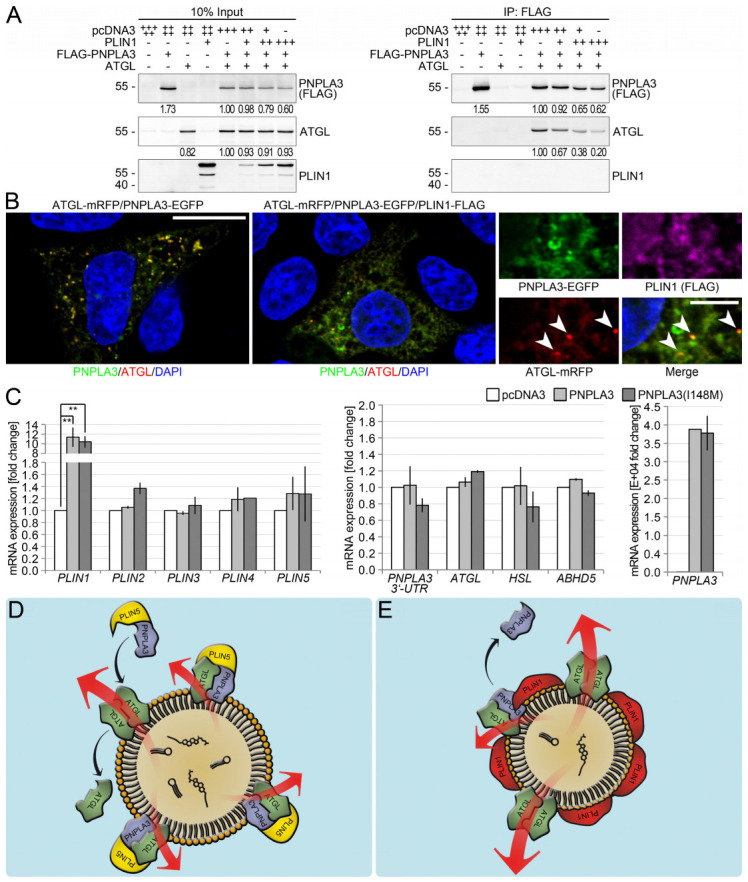
Regulatory effect of PNPLA3 and PLIN1. (**A**) Immunoblot analysis of PNPLA3, ATGL and perilipin 1 in co-immunoprecipitates of FLAG-tagged PNPLA3 in HEK293T-cells transfected with FLAG-tagged PNPLA3 and ATGL alone and together with increasing amounts of PLIN1. Although perilipin 1 does not interact with PNPLA3 or ATGL, its presence causes a disruption of the PNPLA3-ATGL-complex. (**B**) Immunofluorescence microscopy of ATGL with PNPLA3 alone or in the presence of perilipin 1 in HepG2-cells transfected with mRFP-tagged ATGL (red) and EGFP-tagged PNPLA3 (green) alone or together with FLAG-tagged PLIN1 (violet; nuclei: DAPI, blue). The presence of perilipin 1 induces translocation of PNPLA3 from LDs to the cytoplasm and reduces the number of ATGL-positive foci. Arrowheads indicate residual ATGL/PNPLA3-double positive foci. (**C**) Real-time PCR analysis of Huh7-cells transfected with PNPLA3, PNPLA3(I148M) or empty vector. Overexpression of either PNPLA3 (*p* = 0.0021) or PNPLA3(I148M) (*p* = 0.0014) leads to a significant induction of PLIN1. Molecular weight (kDa) is given on the left. All bars represent the mean ± SD. *p* < 0.05 (*); *p* ≤ 0.01 (**). The results of a representative experiment are shown. Experiments were repeated 3 times with comparable results. (**D**,**E**) Working model. (**D**) Perilipin 5 (PLIN5) recruits PNPLA3 to the lipolytically active ATGL-complex forming a trimeric complex. PNPLA3 displaces ATGL from the complex and thereby markedly reduces lipolytic activity. The equilibrium between breakdown and expansion of LDs is shifted towards their expansion. This negative influence of PNPLA3 on the overall lipolytic activity is further enhanced by PNPLA3(I148M). (**E**) Perilipin 1 displaces PNPLA3 from the complex, thus allowing di- or oligomerization of ATGL. In the absence of PNPLA3 as part of the lipolytically active ATGL-complex, the activity is significantly higher, so that the degradation of triacylglycerides and sterol esters is elevated, and LDs become smaller. In carriers of the PNPLA3(I148M) polymorphism, the perilipin 1-mediated regulatory mechanism that displaces PNPLA3 from LDs appears to be disturbed.

To investigate the impact of perilipin 1 on the stability of the PNPLA3-ATGL-complex, co-IP and subsequent immunoblot were carried out. Increasing amounts of perilipin 1 led to a dose-dependent release of up to 80% of PNPLA3 from the PNPLA3–ATGL complex (Figure 6A).

Cells co-expressing perilipin 1 and PNPLA3 exhibited granular, cytoplasmic localization for PNPLA3, which was negative for ATGL, whereas LDs were positive for perilipin 1 but not PNPLA3 (Figure 6B). In addition, the number of ATGL-positive foci that represented the small residual LDs were strongly reduced (Figure A7). In contrast, when the lipolytic activity of ATGL was inhibited, the number of such foci increased. With normal ATGL-activity, no ATGL-positive foci were detectable and only diffused cytoplasmic localization was visible (Figure 5A). The perilipin 1-mediated displacement of PNPLA3 from the PNPLA3-ATGL complex appeared to reduce the negative impact of PNPLA3 on ATGL and, therefore, significantly increased ATGL-mediated lipolysis.

Finally, the overexpression of PNPLA3 or PNPLA3(I148M) was examined to determine the effects on the expression of perilipin 1–5, the lipases ATGL, HSL, and PNPLA3, as well as the co-activator of ATGL, ABHD5. Overexpression of either PNPLA3 or PNPLA3(I148M) strongly induced perilipin 1 irrespective of cell type (Figure 6C), pointing to a possible feedback mechanism in which PNPLA3 induces perilipin 1 that replaces PNPLA3 from LDs (Figure 6D,E).

## 4. Discussion

Our comprehensive in situ and in vitro data provide evidence for the pivotal role of deregulated basal lipolysis in the progression of steatosis to steatohepatitis. In this process, we could demonstrate that PNPLA3 competes with ATGL for perilipin 5 binding within lipolytic perilipin 5-ATGL complexes. We are the first to show that PNPLA3 physically interacts with perilipin 5 and ATGL at larger LDs found in carriers of the PNPLA3(I148M) polymorphism. In addition, we are the first to be able to localize PNPLA3 in a large collective of patients with steatohepatitis in human liver biopsies in situ with respect to different PNPLA3-polymorphisms. We demonstrated that PNPLA3 competitively displaces ATGL from LDs and thereby strongly reduced the lipolytic activity, thereby favouring LD-accumulation. Both perilipins 1 and 5 have been shown to be key regulators of lipolysis in different tissues. In adipocytes, perilipin 1 regulates lipolysis via HSL and ABHD5, whereas in cell types using oxidative energy supply, perilipin 5 [31] controls lipolysis by interacting with ATGL and ABHD5 [41]. Strikingly, co-expression of PNPLA3 and perilipin 5 led to an increased association of both proteins with LDs. Although perilipin 1 did not interact with PNPLA3, co-expression resulted in an almost complete displacement of PNPLA3 from LDs. We hypothesize that perilipin 1 may not function as a LD-barrier protecting against degradation of TAGs [42], but rather as a factor displacing PNPLA3 from the PNPLA3-ATGL-complex, thus reducing the negative impact of PNPLA3 on ATGL-mediated lipolysis and switching to an alternative type of lipolysis.

We have demonstrated a possible pathophysiologic mechanism of how the polymorphism I148M of the lipase PNPLA3 (for epidemiologic studies see [6,7,8]) may trigger NAFLD-progression on the molecular level. We and others have shown that PNPLA3(I148M) leads to a reduction in the lipolytic activity and an accumulation of TAGs in LDs of increased sizes via an indirect effect of PNPLA3 on ATGL-mediated lipolytic activity [9,10,13,43]. Pnpla3 knockout mice did not develop steatosis [11,12]. In contrast, a liver specific knockin or overexpression of PNPLA3(I148M) in mice induced FLD, not observed for PNPLA3 [13,14]. Consistent with these findings, the presence of PNPLA3 led to a marked reduction in ATGL-mediated lipolytic activity with significantly increased LDs, an effect even stronger for PNPLA3(I148M). We have shown that the mechanism for the reduced amount of lipolytically active ATGL at LDs may be due to the disruption of homodimeric or -multimeric complexes formed by ATGL by displacing ATGL in a perilipin 5-dependent manner. This displacement, mediated by an interaction of PNPLA3 with ATGL, was also observed by Wang et al. [18]. Strikingly, this suggests that this weak interaction [18] could be dramatically increased in the presence of perilipin 5 and decreased by perilipin 1, indicating opposing roles of perilipin 1 and 5 under these conditions. Sequestration of ABHD5 by PNPLA3 has been shown to prevent activation of ATGL, yet only a very weak interaction could be demonstrated [18,44]. Both the weak negative influence of PNPLA3 and the strong negative influence of PNPLA3(I148M) on ATGL-mediated lipolytic activity have been recently demonstrated in Huh7 cells [18]. However, this reduction in total lipolytic activity could not be explained by the loss of lipolytic activity of PNPLA3 caused by the missense mutation [10] since PNPLA3 exhibits only low lipolytic activity compared to ATGL and the loss of this low activity does not provide a sufficient explanation for the strong phenotype in PNPLA3(I148M)-carriers. Two possible mechanisms may lead to a reduction in total lipolytic activity by PNPLA3: First, PNPLA3 may stoichiometrically compete with ATGL for LD-binding and thus easily block potential binding sites for ATGL without the interaction of both proteins. Second, PNPLA3 may interact directly with ATGL, thus affecting the activity of ATGL. An interaction between PNPLA3 and ABHD5, was proposed as a model for brown adipocytes, in which PNPLA3 sequesters ABHD5 and therefore prevents binding and activation of ATGL, thereby indirectly suppressing lipolysis [44]. A similar mechanism in suppressing lipolysis by sequestration of ABHD5 was shown for perilipins 1 and 5. In livers of untreated mice with ATGL deletion, we could only observe slightly increased perilipin 5, ABHD5, and PNPLA3 levels, with respect to the control mice. Interestingly, in the livers of hepatocyte-specific perilipin 2 knockout mice treated with an HFD and CDAA diet, leading to steatosis and steatohepatitis, upregulation of perilipin 5 was observed concomitant to a decrease in inflammation, cell death and fibrosis (parallel manuscript in preparation).

Our findings demonstrate important implications for the mechanism by which PNPLA3(I148M) promotes progression to steatohepatitis in FLD, irrespective of the causative agent. We hypothesize that cell stress caused by supernutrition, alcohol or HCV together with a block of basal lipolysis caused by PNPLA3(148M) leads to hepatocyte ballooning and inflammation. In ballooned hepatocytes, the hallmark of steatohepatitis, perilipin 1 was markedly reduced, whereas perilipin 2 was strongly expressed but localized diffusely in the cytoplasm. Cytoplasmic localization of perilipin 2 in ballooned hepatocytes is a phenomenon that has not been observed in any other cell type under physiological conditions and can therefore be interpreted as a sign of a severely damaged/dying cell.

Our study identified several key players that are dysregulated in steatohepatitis and therefore may have both a diagnostic and a therapeutic potential. Generally, the presence of ballooned hepatocytes in non-heavy drinkers indicates a heterozygous or homozygous PNPLA3(I148M)-mutation [35]. The antibody staining of accumulated PNPLA3 at LDs in situ [9] in PNPLA3(I148M) carriers may be used diagnostically. The identification of altered ATGL activity makes this mechanism a potential drug target and the therapeutic intervention may be guided based on the PNPLA3-status of the patient, thus making this method applicable for personalized therapy. Since perilipin 1 is strongly downregulated in severe ASH and at the cellular level in ballooned hepatocytes, and since we could unravel under in vitro conditions that perilipin 1 is able to replace PNPLA3 from the ATGL-complex, it is possible that perilipin 1 may also be a possible therapeutic target in steatohepatitis in patients with PNPLA3(I148M).

## 5. Conclusions

With this study involving human, mouse and cell culture, we could unravel the LD-associated protein perilipin 5 as a binding partner of PNPLA3 and ATGL in vitro and in situ, and the functional role of perilipin 5 ATGL/PNPLA3 complex formation during steatohepatitis, especially in the frequent PNPLA3 polymorphism I148M. Our data point towards an important role of deregulated lipolysis during the formation of ballooned cells in steatohepatitis, triggering the progression of inflammation and fibrosis.

## Figures and Tables

**Table 1:** Patient characteristics.

	*PNPLA3* Status	Number of Cases	Sex (% Male)	Age	Brunt-Score (1–3)	Ballooning (0–2)	NAS-Score (0–7)	Inflammation (0–3)	Fibrosis (% Cirrhosis)
NASH	*C*/*C*	7	42.9	54 ± 14	2.00 ± 0.58	1.14 ± 0.38	4.83 ± 1.47	1.00 ± 0.58	14.3
	*C*/*G*	10	50	48 ± 11	2.00 ± 0.00	1.40 ± 0.52	4.78 ± 1.30	1.50 ± 0.71	20.0
	*G*/*G*	6	50	43 ± 15	2.67 ± 0.52	1.00 ± 0.00	4.43 ± 1.51	1.00 ± 0.63	0.0
	*C*/*C*	6	66.7	54 ± 10	1.50 ± 0.84	1.83 ± 0.41	6.00 ± 1.26	2.67 ± 0.52	83.3
ASH	*C*/*G*	8	75	46 ± 12	2.50 ± 0.76	1.88 ± 0.35	6.50 ± 1.20	2.25 ± 0.46	88.9
	*G*/*G*	1	100	70	2.00	2.00	7.00	3.00	100
	*C*/*C*	2	0	64 ± 13	2.00 ± 1.41	1.50 ± 0.71	5.00 ± 2.83	1.50 ± 0.71	50.0
BASH	*C*/*G*	1	0	59	1.00	2.00	5.00	2.00	100.0
	*G*/*G*	1	100	31	3.00	2.00	6.00	1.00	0.0
	*C*/*C*	3	33.3	52 ± 7	1.00 ± 0.00	0.67 ± 0.58	2.67 ± 0.58	1.00 ± 0.00	33.0
HCV	*C*/*G*	3	0	66 ± 9	1.00 ± 0.00	1.00 ± 0.00	3.00 ± 0.00	1.00 ± 0.00	66.0
	*G*/*G*	3	33.3	53 ± 6	1.33 ± 0.58	0.67 ± 0.58	3.00 ± 2.00	1.00 ± 1.00	100
	*C*/*C*	1	100	n.a.	1.00	0.00	1.00	0.00	0.0
Control	*C*/*G*	1	n.a.	n.a.	1.00	0.00	0.00	0.00	0.0
	*G*/*G*	1	n.a.	14	2.00	0.00	4.00	1.00	0.0
Unclear	*C*/*C*	2	100	46 ± 0	2.50 ± 0.71	1.00 ± 0.00	5.00 ± 1.41	1.50 ± 0.71	50.0
	*C*/*G*	2	50	19 ± 2	2.50 ± 0.71	1.50 ± 0.71	5.00 ± 0.00	1.00 ± 0.00	0.0
	n.a.	1	100	34	3.00 ± 0.00	1.00 ± 0.00	5.00	1.00 ± 0.00	0.0

Summary of the clinical parameters of the human liver samples analyzed. Values are given as mean ± SD.

**Table 2:** Histopathology of liver biopsies with respect to *PNPLA3*-status.

	*PNPLA3* Status	Steatosis (%)	Perilipin 1 (0–3)	Perilipin 2 (0–3)	Perilipin 3 (0–3)	Perilipin 4 (0–3)	Perilipin 5 (0–3)
NASH	*C*/*C*	43.6 ± 20.6	2.50 ± 0.29	2.71 ± 0.27	0.79 ± 0.27	0.79 ± 0.27	1.07 ± 0.19
	*C*/*G*	48.5 ± 11.3	2.55 ± 0.64	2.70 ± 0.26	0.95 ± 0.16	0.70 ± 0.26	1.30 ± 0.63
	*G*/*G*	65.0 ± 21.5	2.08 ± 0.38	2.58 ± 0.38	0.92 ± 0.20	0.67 ± 0.41	1.17 ± 0.41
	*C*/*C*	29.2 ± 27.1	0.83 ± 0.61	2.50 ± 0.45	2.17 ± 0.41	2.00 ± 0.00	2.00 ± 0.00
ASH	*C*/*G*	56.3 ± 22.5	1.19 ± 0.46	2.72 ± 0.53	2.00 ± 0.38	2.00 ± 0.00	2.00 ± 0.00
	*G*/*G*	35.0	1.00	2.50	1.50	2.00	2.00
	*C*/*C*	52.5 ± 38.9	0.75 ± 0.35	2.75 ± 0.35	1.25 ± 0.35	2.00 ± 0.00	2.00 ± 0.00
BASH	*C*/*G*	25.0	2.00	2.00	1.50	2.00	2.00
	*G*/*G*	80.0	0.50	3.00	2.50	2.00	2.00
	*C*/*C*	18.3 ± 7.6	1.16 ± 0.29	2.00 ± 0.00	1.50 ± 0.00	2.00 ± 0.00	2.00 ± 0.00
HCV	*C*/*G*	16.7 ± 12.6	1.26 ± 0.76	2.33 ± 0.29	1.50 ± 0.00	2.00 ± 0.00	2.00 ± 0.00
	*G*/*G*	23.3 ± 12.6	1.16 ± 0.29	2.33 ± 0.29	1.50 ± 0.00	2.00 ± 0.00	2.00 ± 0.00
	*C*/*C*	15.0	2.00	3.00	2.00	2.00	2.00
Control	*C*/*G*	5.0	1.50	1.50	1.50	2.00	2.00
	*G*/*G*	60.0	2.00	2.50	1.00	2.00	2.00
Unclear	*C*/*C*	40.0 ± 21.0	1.50 ± 0.71	2.75 ± 0.35	1.75 ± 0.35	2.00 ± 0.00	2.00 ± 0.00
	*C*/*G*	42.5 ± 38.9	1.50 ± 0.71	3.00 ± 0.00	1.50 ± 0.00	2.00 ± 0.00	2.00 ± 0.00
	n.a.	55.0	2.00	3.00	2.50	2.00	2.00

Semiquantitative evaluation of the staining intensity for perilipin 1–5 in human liver samples. Values are given as mean ± SD.

## Data Availability

Data may be made available upon request.

## Supplementary Materials

The following supporting information can be downloaded at: https://www.mdpi.com/article/10.3390/cells12010073/s1, Table S1. Oligonucleotides for amplification and subcloning of different genes in pcDNA3; Table S2. Oligonucleotides to introduce PNPLA3 sequence variant; Table S3. Oligonucleotides for quantitative real-time PCR; Table S4. Antibodies [45].
