# Supplementary material for: PNPLA3(I148M) Inhibits Lipolysis by Perilipin-5-Dependent Competition with ATGL
Source: Cells. 2022 Dec 24;12(1):73. doi: 10.3390/cells12010073 (PMC9818421; doi:10.3390/cells12010073)
Supplement: Supplementary file 1 [file cells-12-00073-s001.zip › cells-2103864-supplementary.pdf]

Supplementary Information

Supplementary Material and Methods

Evaluation of immunohistochemistry

Stained sections were digitised with a NanoZoomer 2.0 HT (Hamamatsu) at a magnification of 40x and the intensity of the staining was quantified using the digital image analysis software QuPath (v.0.1.2) [1]. First, image type was set to brightfield (H-DAB). After this, using the rectangle tool, an annotation was placed on the entire stained biopsy including the area without tissue. For the annotated area, the staining intensity was normalized via preprocessing, estimate stain vector on auto mode. The wand tool was used to annotate the entire biopsy, excluding portal tracts, vessels and staining artefacts. The number of DAB-positive pixels per μm^2 was then determined via analysis of the positive pixel count.

Quantification of total area of LDs

To determine the area occupied by LDs in biopsies, the digitised image was opened with the software Amperio ImageScope (v.12.2.2.5015). Using the extract region tool, the entire area occupied by the biopsy was extracted and saved as a TIF-file with height and width reduced to 50%. Using Photoshop (CS5 v.12.0) the extracted image was further converted into a black and white image und the colours were inverted so that LDs appeared in black. In addition, portal tracts, vessels, and staining artefacts were removed by erasing and the image was saved as a JPG-file. These files were then opened with ImageJ (v.1.52n), converted into an 8-bit image and the threshold of the image set in a way that all LDs appeared black. Subsequently, with the function "analyze particles" with the following parameters: size (pixel^2): 15-infinity and circularity 0.04-1.00 the particles were analysed. The total area of all LDs counted was then referenced to the total area of the biopsy. The percentage of the total area occupied by the LDs was then calculated based on the total area of the biopsy.

Quantification of LD-size

High-resolution images were taken with a confocal microscope. In addition to LD-staining with BODIPY, co-staining with PNPLA3, ATGL, and other LD-associated proteins was performed, using overexposure to detect the exact dimensions of a positively transfected cell. Image files were then opened with ImageJ (v.1.52n), converted into an 8-bit image and the threshold of the image set in a way that all LDs appeared black. With the polygon selection tool, the dimension of the positive transfected cells was outlined and the LDs within this area were quantified with the function "analyze particles" with the following parameters: size (pixel^2): 0.05-infinity and circularity 0.00-1.00.

LD-quantification of CRISPR single-cell clones (HEK293T) was not possible with the method described above, therefore, confocal images of the nuclei as well as LDs were converted with ImageJ into an 8-bit image and the threshold of the image set in a way that all LDs and nuclei appeared black. The total area of the LDs and that of the nuclei was measured with the function "analyze particles" with the following parameters: size (pixel^2): 0.00-infinity and circularity 0.00-1.00. The ratio between the total area of LDs and the total area of the nuclei was calculated.

Cloning constructs

For the cloning of the eukaryotic expression vectors, a slightly modified protocol described by Oliner [2] was used. The target vector pcDAN3-FLAG was linearized with *HindIII*, *XhoI* and gel purified. Using RNA obtained from human liver, PCR with oligonucleotides for the target genes was carried out after cDNA synthesis. The oligonucleotides were designed to have homology regions to the ends of the cut target vector. The PCR products of the target genes were also gel purified and transformed together with the linearized vector in a ratio of 3:1 (insert:vector) into *E. Coli* Top10 cells.

Table S1. Oligonucleotides for amplification and subcloning of different genes in pcDNA3.

Gene	Sequence [5′-3′]
ATGL	5′-CGACTCACTATAGGGAGACCCAAGCTTGCCACCATGTTTCCCCGCGAGAAGAC-3′
	5′-GTCGTCATCGTCTTTGTAGTCCTCGAGCAGCCCCAGGGCCCCGATCAC-3′
	5′-GTCGTCATCGTCTTTGTAGTCCTCGAGTCACAGCCCCAGGGCCCCGATC-3′
PNPLA3	5′-CGACTCACTATAGGGAGACCCAAGCTTGCCACCATGTACGACGCAGAGCG-3′
	5′-GTCGTCATCGTCTTTGTAGTCCTCGAGCAGACTCTTCTCTAGTGAAAAACTGG-3′
	5′-GTCGTCATCGTCTTTGTAGTCCTCGAGTCACAGACTCTTCTCTAGTGAAAAACTG-3′
ABHD5	5′-CGACTCACTATAGGGAGACCCAAGCTTGCCACCATGGCGGCGGAGGAGGAG-3′
	5′-GTCGTCATCGTCTTTGTAGTCCTCGAGGTCCACAGTGTCGCAGATC-3′
	5′-GTCGTCATCGTCTTTGTAGTCCTCGAGTCAGTCCACAGTGTCGCAG-3′
PLIN1	5′-CGACTCACTATAGGGAGACCCAAGCTTGCCACCATGGCAGTCAACAAAGGCCTC-3′
	5′-GTCGTCATCGTCTTTGTAGTCCTCGAGGCTCTTCTTGCGCAGCTG-3′
	5′-GTCGTCATCGTCTTTGTAGTCCTCGAGTCAGCTCTTCTTGCGCAGC-3′
PLIN2	5′-CGACTCACTATAGGGAGACCCAAGCTTGCCACCATGGCATCCGTTGCAGTTG-3′
	5′-GTCGTCATCGTCTTTGTAGTCCTCGAGATGAGTTTTATGCTCAGATCGC-3′
	5′-GTCGTCATCGTCTTTGTAGTCCTCGAGTTAATGAGTTTTATGCTCAGATCG-3′
PLIN3	5′-CGACTCACTATAGGGAGACCCAAGCTTGCCACCATGTCTGCCGACGGGGCAG-3′
	5′-GTCGTCATCGTCTTTGTAGTCCTCGAGCTTCTTCTCCTCCGGGGCTTTC-3′
	5′-GTCGTCATCGTCTTTGTAGTCCTCGAGCTACTTCTTCTCCTCCGGGGCTTTC-3′
PLIN5	5′-CGACTCACTATAGGGAGACCCAAGCTTGCCACCATGTCTGAAGAAGAGGCGGC-3′
	5′-GTCGTCATCGTCTTTGTAGTCCTCGAGGAAGTCCAGCTCGGGCATC-3′
	5′-GTCGTCATCGTCTTTGTAGTCCTCGAGTCAGAAGTCCAGCTCGGGC-3′

The table shows the forward, reverse and reverse oligonucleotide sequences where the stop codons remain intact (cloning without FLAG tag).

The sequence variant of PNPLA3 was generated by mutagenesis PCR with the following oligonucleotides:

Table S2. Oligonucleotides to introduce PNPLA3 sequence variant.

Gene	Sequence [5′-3′]
PNPLA3 I148M	5′-TTCCTGCTTCATGCCCTTCTACAGT-3′
	5′-ACTGTAGAAGGGCATGAAGCAGGAA-3′

The table shows the sequences of the forward and reverse oligonucleotides used in mutagenesis PCR.

Oligonucleotides used in quantitative real-time PCR

Table S3. Oligonucleotides for quantitative real-time PCR.

Gene	Sequence [5′-3′]
PLIN1	5′-CTGCCGGTGGTGAGTGGCAC-3′
	5′-CACAGAGGCCACCAGGGGGT-3′
PLIN2	5′-GATGGCAGGCGACATCTACT-3′
	5′-GGACCTACCAGCCAGTTGAG-3′
PLIN3	5′-ATCGGTCATGGGCTCCCGCT-3′
	5′-CTGTTCCTGCCGCTGCTGCT-3′
PLIN4	5′-TCGGAAAAGCAGATGGTGTC-3′
	5′-TCCCTGGACCACTCCCTTAG-3′
PLIN5	5′-GCTGTGGATGTTGTACTGGA-3′
	5′-CTGCTGTCTCCTCTGATCCT-3′
ABHD5	5′-TGCAGACTCCAAGTGGTGAG-3′
	5′-TGTCAGGGTGCATTTTACCA-3′
PNPLA3	5′-GAAGTCGTGGATGCCTTGGT-3′

	5′-ATATCGCACGCCTCTGAAGG-3′
PNPLA3 3′-UTR	5′-GCCTCTGAGCTGAGTTGGTT-3′<br>5′-GAGTTAAGTGCTGGACCGCT-3′
ATGL	5′-ACCAGCATCCAGTTCAACCT-3′<br>5′-ATCCCTGCTTGCACATCTCT-3′
HSL	5′-AGCCTTCTGGAACATCACCGAG-3′<br>5′-TCGGCAGTCAGTGGCATCTCAA-3′

The table shows the forward and reverse oligonucleotide sequences for different genes used for quantitative real-time PCR analysis.

Antibodies

Table S4. Antibodies.

Antibody	Species	Type	Reference
Actin	Mouse	Monoclonal	MAB1501; Merck
ATGL	Rabbit	Polyclonal	2138; Cell Signaling
FLAG	Mouse	Monoclonal	F3165; Sigma-Aldrich
HA	Mouse	Monoclonal	2367; Cell Signaling
Perilipin 1	Mouse	Monoclonal	PERI 112.17; Progen
Perilipin 1	Guinea pig	Polyclonal	GP29, Progen
Perilipin 2	Mouse	Monoclonal	AP125; Progen
Perilipin 3	Guinea pig	Polyclonal	GP30, Progen
Perilipin 4	Guinea pig	Polyclonal	GP38, Progen
Perilipin 5	Guinea pig	Polyclonal	GP31, Progen
Phospho-HSL (Ser660)	Rabbit	Polyclonal	4126; Cell Signaling
PNPLA3	Rabbit	Polyclonal	SAB2108057, lot: QC56471; Sigma-Aldrich
PNPLA3	Mouse	Polyclonal	H00080339-B01P, lot: H6091; Abnova

The table shows the antibodies used for immunoblot, immunohistochemistry or immunofluorescence analyses.

1. Bankhead, P.; Loughrey, M.B.; Fernandez, J.A.; Dombrowski, Y.; McArt, D.G.; Dunne, P.D.; McQuaid, S.; Gray, R.T.; Murray, L.J.; Coleman, H.G.; et al. QuPath: Open source software for digital pathology image analysis. *Sci Rep* **2017**, *7*, 16878, doi:10.1038/s41598-017-17204-5.
2. Oliner, J.D.; Kinzler, K.W.; Vogelstein, B. In vivo cloning of PCR products in E. coli. *Nucleic Acids Res* **1993**, *21*, 5192-5197, doi:10.1093/nar/21.22.5192.
